# Hidrox^®^ and Endometriosis: Biochemical Evaluation of Oxidative Stress and Pain

**DOI:** 10.3390/antiox10050720

**Published:** 2021-05-04

**Authors:** Marika Cordaro, Angela Trovato Salinaro, Rosalba Siracusa, Ramona D’Amico, Daniela Impellizzeri, Maria Scuto, Maria Laura Ontario, Livia Interdonato, Roberto Crea, Roberta Fusco, Salvatore Cuzzocrea, Rosanna Di Paola, Vittorio Calabrese

**Affiliations:** 1 Department of Biomedical, Dental and Morphological and Functional Imaging University of Messina, Via Consolare Valeria, 98125 Messina, Italy; cordarom@unime.it (M.C.); dipaolar@unime.it (R.D.P.); calabres@unict.it (V.C.); 2 Department of Biomedical and Biotechnological Sciences, University of Catania, 95124 Catania, Italy; trovato@unict.it (A.T.S.); mary-amir@hotmail.it (M.S.); marialaura.ontario@ontariosrl.it (M.L.O.); 3 Department of Chemical, Biological, Pharmaceutical and Environmental Sciences, University of Messina, 98166 Messina, Italy; rsiracusa@unime.it (R.S.); rdamico@unime.it (R.D.); dimpellizzeri@unime.it (D.I.); livia.interdonato@yahoo.it (L.I.); 4 Oliphenol LLC., 26225 Eden Landing Road, Unit C, Hayward, CA 94545, USA; robertocrea48@gmail.com

**Keywords:** endometriosis, oxidative stress, pain

## Abstract

Endometriosis is a gynecological and painful condition affecting women of reproductive age. It is characterized by dysfunctional endometrium-like implants outside of the uterine cavity. The purpose of this study was to evaluate the effects of Hidrox^®^, an aqueous extract of olive pulp containing hydroxytyrosol, on endometriotic lesions associated with pro-oxidative alterations and pain-like behaviors. Endometriosis was induced by intraperitoneal injection of uterine fragments, and Hidrox^®^ was administered daily. At the end of the 14-day treatment, behavioral alterations were assessed and hippocampal tissues were collected. Laparotomy was performed, and the endometrial implants were harvested for histological and biochemical analysis. Hidrox^®^ treatment reduced endometriotic implant area, diameter and volumes. Vehicle-treated rats showed lesional fibrosis, epithelial–mesenchymal transition and fibroblast–myofibroblast transdifferentiation, angiogenesis and pro-oxidative alterations in the peritoneal cavity. Hidrox^®^ treatment reduced the aniline blue-stained area, α-smooth muscle actin (α-sma) and CD34 positive expressions. Moreover, it reduced mast cell recruitment into the lesions, myeloperoxidase activity and lipid peroxidation and increased superoxide dismutase (SOD) activity and glutathione levels in the endometrial explants. In the peritoneal fluid, Hidrox^®^ treatment reduced interleukin (IL)-1β, IL2, IL6, tumor necrosis factor-α (TNF-α) and vascular endothelial grow factor (VEGF) levels increased by the disease. Hidrox^®^ administration also reduced peripheral and visceral sensibility as shown by the behavioral tests (open field test, hot plate test, elevated plus maze test and acetic-acid-induced abdominal contractions). Animals treated with Hidrox^®^ also showed reduced blood–brain barrier permeability and mast cell infiltration in the hippocampus, as well as astrocyte and microglia activation and brain oxidative status restoring brain-derived neurotrophic factor (BDNF) protein expression and increasing Nuclear factor erythroid 2-related factor 2 (Nfr2) nuclear translocation. In conclusion, Hidrox^®^ displayed potential ameliorative effects on endometriotic implants and related pain-induced behaviors due to its potent antioxidative properties.

## 1. Introduction

Endometriosis is a debilitating disease that affects 10% of reproductive-age women [1,2]. It induces pelvic-organ dysfunction, infertility and chronic pain, adversely affecting quality of life [3,4,5].

Besides its influence on women’s health, endometriosis is related to an enormous economic burden [6]. Although the cause of endometriosis is undetermined, the principal theory proposes retrograde menstruation as a probable cause [7]. This hypothesis is supported by the evidence that endometrial tissue injection or seeding into the peritoneal cavity of both rodents and baboons can lead to disease growth [8]. Once this tissue is injected into the peritoneal cavity, it must firstly attach, invade the mesothelium, create a vascular supply and proliferate. The peritoneal environment of patients, in fact, is transformed promoting the development of the disease [9]. The most serious clinical type of endometriosis is known as deep endometriosis, and its clinical management is difficult [10,11]. It is characterized by endometrioma stroma and epithelial cells encapsulated in surrounding tissues with smooth muscle metaplasia and exhibiting extensive fibrosis. In particular, endometriotic cells acquire mobility and invasiveness through the epithelial–mesenchymal transition or fibroblast–myofibroblast differentiation increasing cyst volume and establishing vascular supply [12].

Convincing evidence shows that endometriosis progression is related to a pro-oxidative and immune mechanism [13]. Overproduction of reactive oxygen species (ROS) is associated with malignancy transmission and increased proliferation rate [14]. Increased oxidative stress markers have been found in samples from women affected by the disease.

Recently an interesting relationship between endometriosis and pain-like behaviors has been established. Changes in stress-responsive brain areas, principally in the hippocampus, have been related to the pain sensitization. Increased oxidative stress has been described in the hippocampus of endometriosis rats. Therefore, the molecules able to reduce the progress of the pathology and the induced pain sensitization are eligible treatments for the disease.

Many studies report the beneficial effects of food natural phytocomponents and the Mediterranean diet in several oxidative and painful diseases [15,16]. The Mediterranean diet proposes high intake of vegetables, cereals, fruit, legumes and olive oil rich in flavonoids and polyphenols [17]. In particular, olive oil contains a natural compound called hydroxytyrosol, widely described as an antioxidant and free radicals fighter [18]. In recent years its antimicrobial, anti-inflammatory and neuroprotective activity has been reported in different diseases [19]. The antioxidant effect of extra virgin olive oil and hydroxytyrosol oral administration has been shown in the brain [20,21]. Additionally, hydroxytyrosol ameliorated working memory and spatio-cognitive performances. By increasing cellular glutathione (GSH) levels and decreasing lipid peroxidation, extra virgin olive oil and hydroxytyrosol protect the signaling mechanism in hippocampal neurons from oxidative damage [22,23]. Interestingly, hydroxytyrosol increases the expression of the nuclear factor erythroid 2-related factor (Nrf2) preserving cellular redox balance and homeostasis [24]. Our recent studies showed that Hidrox^®^, an aqueous extract of olive pulp containing 40–50% of hydroxytyrosol, prevents the neurodegenerative progression of Parkinson’s disease by managing the Nrf2 pathway and cellular redox homeostasis [25]. Thus, the aim of this study was to evaluate the effect of Hidrox^®^ administration on endometriotic lesions and the associated pro-oxidative and neuropsychiatric symptoms. 

## 2. Materials and Methods

### 2.1. Animals

Female Sprague–Dawley rats (200–250 g, 8–10 weeks old) (Envigo, Milan, Italy) were used in this research. The University of Messina Review Board for animal care (OPBA) approved the study (ethical approval number: 499/2018-PR). All animal experiments agree with the new Italian regulations (D.Lgs 2014/26), EU regulations (EU Directive 2010/63) and the ARRIVE guidelines.

### 2.2. Experimental Protocol

Animals were randomly divided into two groups, donor or recipient, and endometriosis was established as already described [26]. To stimulate similar estrogen levels, donor rats were intraperitoneally injected with 10 international units (IU) pregnant mare serum gonadotropin to induce similar estrogen levels between various animals. The animals were euthanized 41 h later by CO_2_ asphyxia. A midline incision was performed, and the uterus was removed and minced with scissors. Tissue from all donors was pooled, and the recipient animals were injected intraperitoneally with the equivalent of tissue from one uterus in 500 μL of phosphate buffered saline (PBS) along the midventral line. Endometriosis was allowed to develop for seven days.

### 2.3. Experimental Groups

Rats were randomized and assigned to the following groups (*n* = 20):

(1) Vehicle group: Rats were subjected to experimental endometriosis as described above, and vehicle (saline) was administered by gavage on the 7th day and for the next 7 days.
(2) Hidrox^®^ group: Rats were subjected to experimental endometriosis as described above, and Hidrox^®^ (10 mg/kg) was administered by gavage on the 7th day and for the next 7 days.
(3) Sham group: Rats were injected intraperitoneally with 500 μL of PBS without endometrial tissue, and vehicle (saline) was administered by gavage on the 7th day and for the next 7 days.

The dose of Hidrox^®^ was based on previous experiments [25,27].

In order to evaluate endometriotic lesions, rats were sacrificed at 14 days after endometriosis induction [26].

Brain tissues were harvested and laparotomy was performed to collect the endometriotic implants.

Implants were excised from both groups, measured [28,29] and processed for histological and biochemical studies.

### 2.4. Open Field Test

Locomotor activity and exploratory behavior were measured using a squared open field area [29,30]. After 1 min of habituation, each rat was placed into a corner of the area and observed for 5 min. For cleaning the apparatus after each analysis, a solution of 20% ethanol was used. The parameters recorded were: number of animal crossings with four legs (spontaneous locomotion), entries in central square and time spent in the central square (in seconds).

### 2.5. Hot Plate

Hot plate test was used to evaluate pain threshold to thermal stimuli [31,32]. Rats were allowed to walk on the hot plate (53.0 ± 0.1 °C) for up to 45 s.

### 2.6. Elevated Plus Maze Test

The elevated plus maze apparatus [33,34] consisted of two closed arms and two open arms connected by a central square. The rat was placed in the apparatus and allowed to move for 5 min. For cleaning the apparatus after each analysis, a solution of 20% ethanol was used. The number of total entries, entries open arms and the time spent in it were reported as the % of open entries and the % of time in open arms.

### 2.7. Acetic-Acid-Induced Abdominal Contractions

The animals received an intraperitoneal injection of 0.6% acetic acid, and the number of acid-induced writhes was observed for 20 min, starting 5 min after administration [35]. A writhe was defined as a contraction of the abdomen following a stretch of the hind limbs. 

### 2.8. Determination of Reduced Glutathione Levels

The levels of reduced glutathione (GSH) were determined in endometriosis lesions and hippocampi to evaluate the endogenous antioxidant defenses. GSH levels were determined using a microplate reader at 412 nm and expressed as ng/mg wet tissue [36,37]. 

### 2.9. Measurement of Lipid Peroxidation

Lipoperoxidation was estimated in endometriosis lesions and hippocampi using the thiobarbituric acid reactive substances (TBARS) test [38,39]. The levels of malondialdehyde (MDA) were determined using a microplate reader at 535 nm and expressed as μmol/mg wet tissue.

### 2.10. Measurement of Superoxide Dismutase (SOD) Activity

In endometriosis lesions and hippocampi determination of SOD activity was performed according to a previously described method [40,41,42]. SOD activity (U/μg protein) was determined using a microplate reader at 560 nm. 

### 2.11. Analysis of Myeloperoxidase (MPO) Activity

Myeloperoxidase activity with 3,30,5,50-Tetramethylbenzidine (TMB) was measured in endometriosis lesions and hippocampi as already described [43,44]. Absorbance was measured at 450 nm to estimate MPO activity.

### 2.12. Enzyme-Linked Immunosorbent Assay

Peritoneal fluid and endometriotic lesions were collected. Interleukin (IL) 10, IL6, tumor necrosis factor (TNF) -α, IL-1β and IL2 levels were determined using an ELISA kit (BioLegend, San Diego, California; R&D Systems, Milan, Italy) [45,46].

### 2.13. Histological Examination

For histopathological investigations, endometriosis lesions were fixed at room temperature in buffered formaldehyde solution (10% in PBS); histological sections were stained with H&E and evaluated using a Leica DM6 microscope (Leica Microsystems SpA, Milan, Italy) equipped with a motorized stage and associated with Leica LAS X Navigator software (Leica Microsystems SpA, Milan, Italy) [47]. Histopathologic scores were evaluated with the formula P (persistence of epithelial cells in the explants) × I (intensity of glands) as described previously [48]: P: 3 = well-preserved epithelial layer, 2 = moderately preserved epithelium with leukocyte infiltrating, 1 = poorly preserved epithelium (occasional epithelial cells only), and 0 = no epithelium; I: from 0 (no glands) to 3 (abundant glands). Additionally, lesion volume was calculated according to the formula: V = (length × width^2^) × 0.5. [48]. The degree of fibrosis was evaluated by the Masson trichrome staining performed according to the manufacturer’s protocol (Bio-Optica, Milan, Italy) [49,50]. Mast cell analyses were performed by toluidine blue staining [51]. 

### 2.14. Immunohistochemical Analysis 

Immunohistochemical localization of α-smooth muscle actin (α-sma), CD34, vascular endothelial grow factor (VEGF) and Ki67 was performed in endometriosis lesions as already described [52]. The sections were incubated overnight with primary antibodies: anti-α-sma antibody (CGA7, Santa Cruz Biotechnology, Heidelberg, Germany), anti-CD34 antibody (sc-74499, Santa Cruz Biotechnology, Heidelberg, Germany), anti-VEGF antibody (sc-7269, Santa Cruz Biotechnology, Heidelberg, Germany) and anti-Ki67 antibody (sc-23900, Santa Cruz Biotechnology, Heidelberg, Germany). All sections were washed with PBS and then treated as previously reported [53]. Stained sections were observed using a Leica DM6 microscope (Leica Microsystems SpA, Milan, Italy) following a typical procedure [54]. The histogram profile is related to the positive pixel intensity value obtained [55].

### 2.15. Western Blot Analysis

Cyst samples and hippocampi were homogenized and Western blots were performed as already described [56]. Specific primary antibody anti- brain-derived neurotrophic factor (BDNF) (ab108319, Abcam, Milan, Italy) or anti-glial fibrillary acidic protein GFAP (sc-33673, Santa Cruz Biotechnology, Heidelberg, Germany) or anti-iba-1 (sc-32725, Santa Cruz Biotechnology, Heidelberg, Germany) or anti-Occludin (sc-133256, Santa Cruz Biotechnology, Heidelberg, Germany) or anti-Claudin-5 (Santa Cruz Biotechnology, sc-374221, Heidelberg, Germany) or anti-Nrf2 (Santa Cruz Biotechnology, sc-365949, Heidelberg, Germany) or anti-Bcl-2 (Santa Cruz Biotechnology, sc-7382, Heidelberg, Germany) or anti-Bax (Santa Cruz Biotechnology, sc-7480, Heidelberg, Germany) was mixed in 5% *w*/*v* nonfat dried milk solution and was incubated at 4 °C overnight. Afterward, blots were incubated with peroxidase-conjugated bovine antimouse IgG secondary antibody or peroxidase-conjugated goat antirabbit IgG (Jackson Immuno Research, West Grove, PA, USA) for 1 h at room temperature [57]. To verify the equal amounts of protein, membranes were also incubated with the antibody against β-actin or lamin A/C (Santa Cruz Biotechnology, Heidelberg, Germany). Signals were detected with enhanced chemiluminescence detection system reagent (Super-Signal West Pico Chemiluminescent Substrate, Pierce, Monza, Italy) [58]. The relative expression of the protein bands was quantified by densitometry with Bio-Rad ChemiDoc XRS software (Bio-Rad, Milan, Italy) and standardized to β-actin or lamin A/C levels. Images of blot signals were imported to analysis software (v2003, Image Quant TL, Amersham Biosciences, Freiburg, Germany) [59].

### 2.16. Statistical Evaluation

All values are expressed as mean ± standard error of the mean (SEM) of *N* observations. For in vivo studies, *N* represents the number of animals used. The results were analyzed by *t*-test when comparing two groups while we used the one-way ANOVA followed by a Bonferroni post hoc test for multiple comparisons. A *p*-value of less than 0.05 was considered significant. * *p* < 0.05 vs. sham, # *p* < 0.05 vs. vehicle, ** *p* < 0.01 vs. sham, ## *p* < 0.01 vs. vehicle, *** *p* < 0.001 vs. sham, ### *p* < 0.001 vs. vehicle.

## 3. Results

### 3.1. Effect of Hidrox^®^ Treatment on Lesion Size in Endometriosis

**Figure 1:**
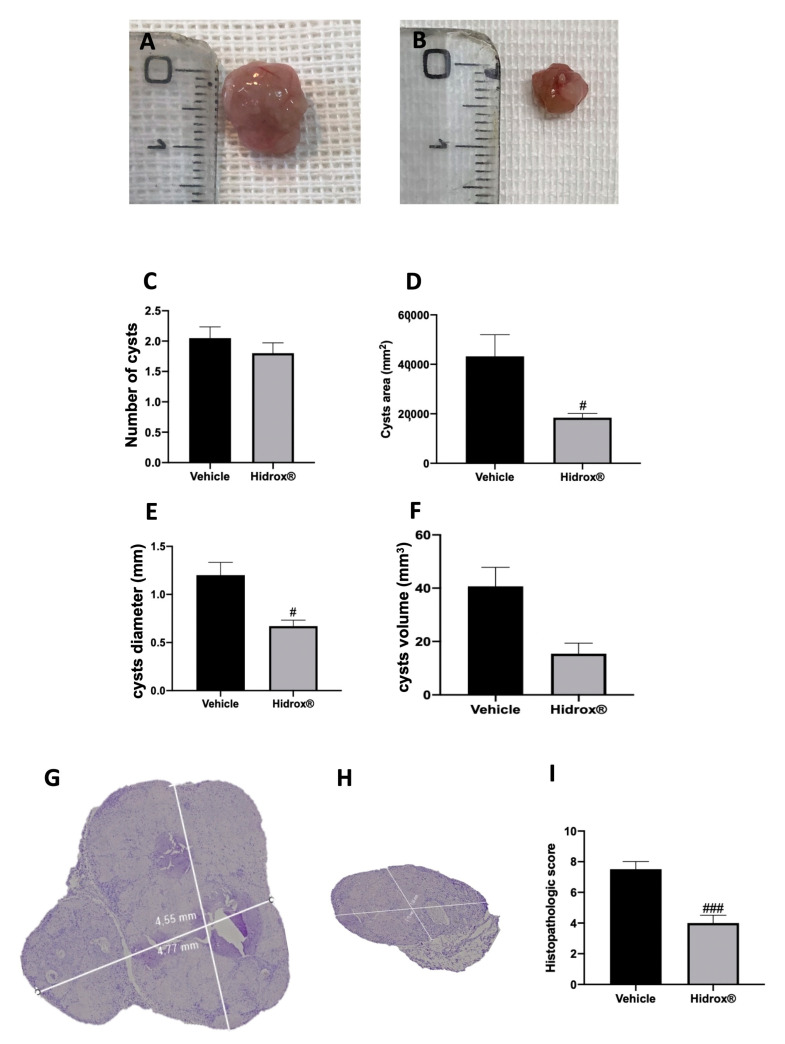
Hidrox^®^ administration reduced lesion size endometriosis-induced: macroscopic analysis: vehicle (**A**), Hidrox^®^ (**B**), cyst number (**C**), cyst area (**D**), cyst diameter (**E**), cyst volume (**F**); histological analysis: vehicle (**G**), Hidrox^®^ (**H**), histopathological score (**I**). For the macroscopic analyses, *n* = 20 animals from each group were employed. For the histological analyses, *n* = 5 animals from each group were employed. A *p*-value of less than 0.05 was considered significant. # *p* < 0.05 vs. vehicle, ### *p* < 0.001 vs. vehicle.

At 14 days of induction, all animals from the vehicle and Hidrox^®^ groups displayed endometriosis lesions, while sham animals did not show any implants. Even the groups did not show different cyst numbers (Figure 1C); the area (Figure 1D), diameter (Figure 1E) and volume (Figure 1F) were smaller in Hidrox^®^ treated animals (Figure 1B) compared to the vehicle (Figure 1A). Histologically, endometriotic lesions from vehicle-treated rats showed abundant stromal structure and endometrial-type glands (Figure 1G,I). Hidrox^®^ administration reduced the histopathological marks of endometriosis (Figure 1H,I).

### 3.2. Effect of Hidrox^®^ Treatment on Fibrosis and Angiogenesis Associated with Endometriosis

**Figure 2:**
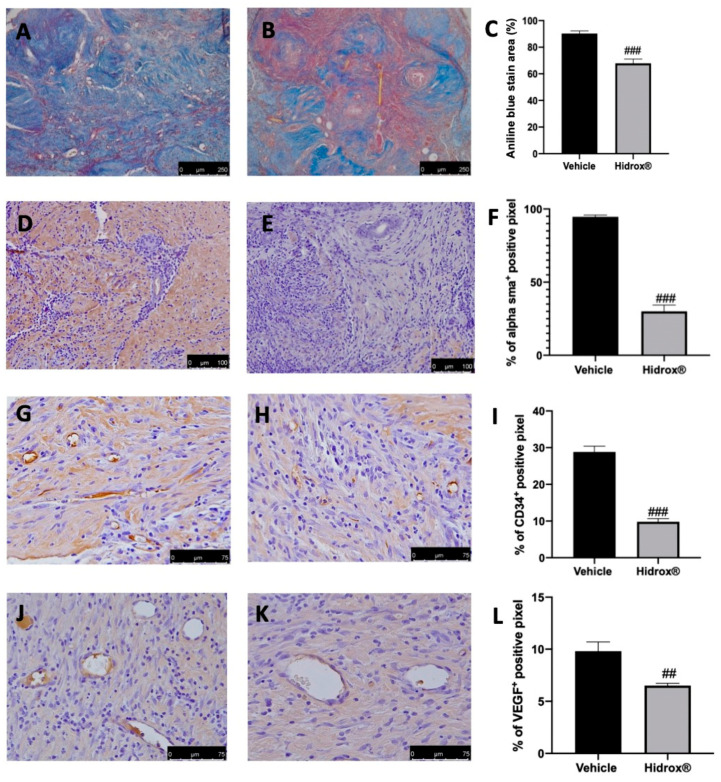
Hidrox^®^ administration reduced fibrosis and angiogenesis endometriosis-induced: Masson trichrome staining: vehicle (**A**), Hidrox^®^ (**B**), aniline blue stain area (**C**); immunohistochemical analysis of α-sma: vehicle (**D**), Hidrox^®^ (**E**), graphical quantification of α-sma expression (**F**); immunohistochemical analysis of CD34: vehicle (**G**), Hidrox^®^ (**H**), graphical quantification of CD34 expression (**I**). Immunohistochemical analysis of VEGF: vehicle (**J**), Hidrox^®^ (**K**), graphical quantification of CD34 expression (**L**). For the analyses, *n* = 5 animals from each group were employed. A *p*-value of less than 0.05 was considered significant. ## *p* < 0.01 vs. vehicle, ### *p* < 0.001 vs. vehicle.

As the advanced stages of endometriosis lesion development are associated with a high degree of tissue fibrosis and increased neovascularization events, we first analyzed the number of collagen fibers, the expression of α-sma as markers for fibrosis. Further, the angiogenesis was evaluated by the abundance of hematopoietic CD34+ stem cells and VEGF expression. The degree of fibrosis was evaluated by Masson’s trichrome staining and α-sma immunolocalization. The collagen fibers were significantly reduced by Hidrox^®^ treatment (Figure 2B,C) as compared to the vehicle-treated animals (Figure 2A,C). α-sma immunolocalization was weakest in lesions from Hidrox^®^ administered animals (Figure 2E,F) as compared to the vehicle rats (Figure 2D,F). Positive immunostaining for CD34 and VEGF was detected in the endometriotic lesions from vehicle-treated rats (Figure 2G,I,J,L), which was decreased by the Hidrox^®^ treatment (Figure 2H,I,K,L).

### 3.3. Effect of Hidrox^®^ Treatment on Hyperproliferation and Anti-Apoptosis

**Figure 3:**
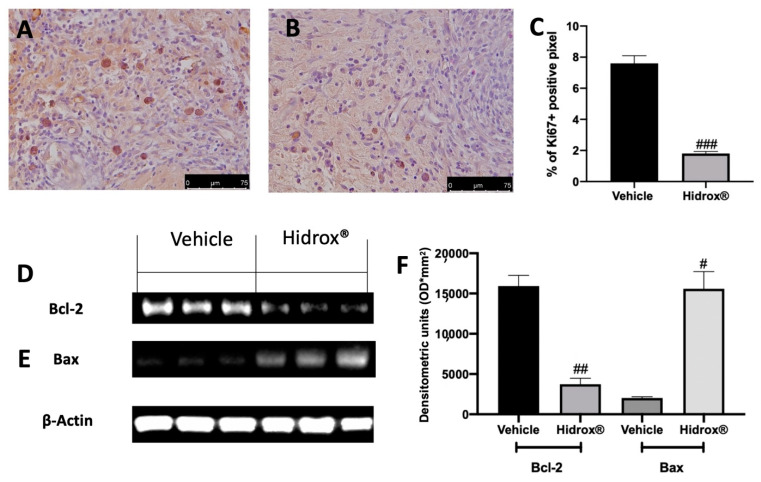
Hidrox^®^ administration reduced hyperproliferation and increased apoptosis endometriosis-induced: immunohistochemical analysis of Ki67: vehicle (**A**), Hidrox^®^ (**B**), graphical quantification of Ki67 expression (**C**), Western blot analysis of Bcl-2 (**D**), Bax (**E**), densitometric analysis (**F**). For the analyses, *n* = 5 animals from each group were employed. A *p*-value of less than 0.05 was considered significant. # *p* < 0.05 vs. vehicle, ## *p* < 0.01 vs. vehicle, ### *p* < 0.001 vs. vehicle.

Important characteristics of endometriosis are hyperproliferation and inhibited apoptosis. We evaluated, using immunohistochemical analysis of the expression of the cell proliferation marker (Ki67) and by Western blot analysis, the anti-apoptotic protein Bcl-2 and the pro-apoptotic protein Bax expressions in the endometriosis lesions. Elevated Ki67 expressions were detected in samples collected from vehicle-treated rats (Figure 3A,C), while Hidrox^®^ administration reduced Ki67 positive staining (Figure 3B,C). Western blot analysis showed elevated Bcl-2 expression and low Bax expression in tissues harvested from vehicle-treated rats, while Hidrox^®^ treatment reduced Bcl-2 (Figure 3D,F) and increased Bax levels (Figure 3E,F).

### 3.4. Effect of Hidrox^®^ Treatment on Mast Cell Number and on Biochemical Parameters

Several papers described the key role of inflammatory cell recruitment at the lesion site during endometriosis and the impaired oxidant–antioxidant balance during the pathology.

**Figure 4:**
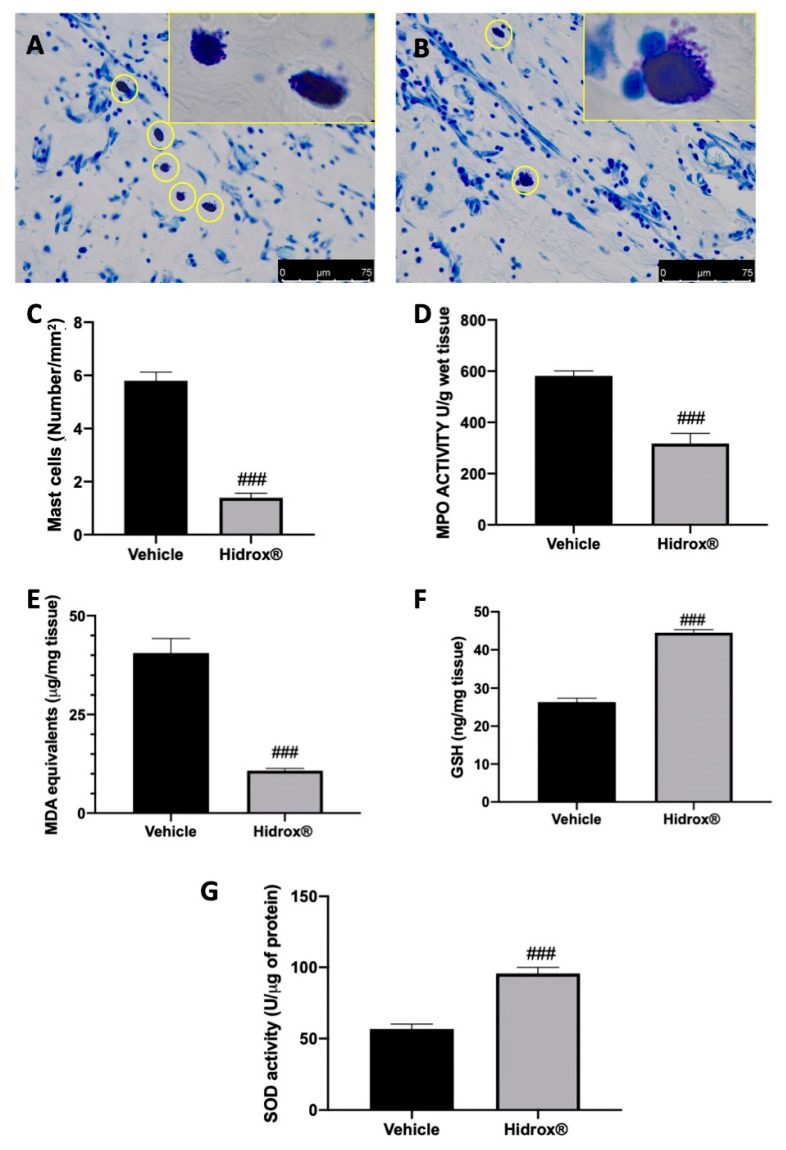
Hidrox^®^ administration reduced mast cell number and pro-oxidative alterations in endometrial explants: Toluidine blue staining of explanted lesions: vehicle (**A**), Hidrox^®^ (**B**), mast cell number (**C**), MPO activity (**D**), MDA levels (**E**), GSH levels (**F**), SOD activity (**G**). For the analyses, *n* = 5 animals from each group were employed. A *p*-value of less than 0.05 was considered significant. ### *p* < 0.001 vs. vehicle.

Toluidine blue staining showed elevated mast cell number in lesions harvested from vehicle-treated rats (Figure 4A,C). Hidrox^®^ treatment reduced mast cell infiltration into the cysts (Figure 4B,C). Levels of GSH and MDA and MPO and SOD activities were determined in endometrial explants. Treatment with Hidrox^®^ resulted in a significant reduction of MPO activity (Figure 4D) and MDA levels (Figure 4E) compared to the vehicle-treated rats. Moreover, Hidrox^®^ administration increased GSH levels (Figure 4F) and SOD activity (Figure 4G).

### 3.5. Effect of Hidrox^®^ Treatment on Cytokine Expressions in Lesions and Peritoneal Fluid 

**Figure 5:**
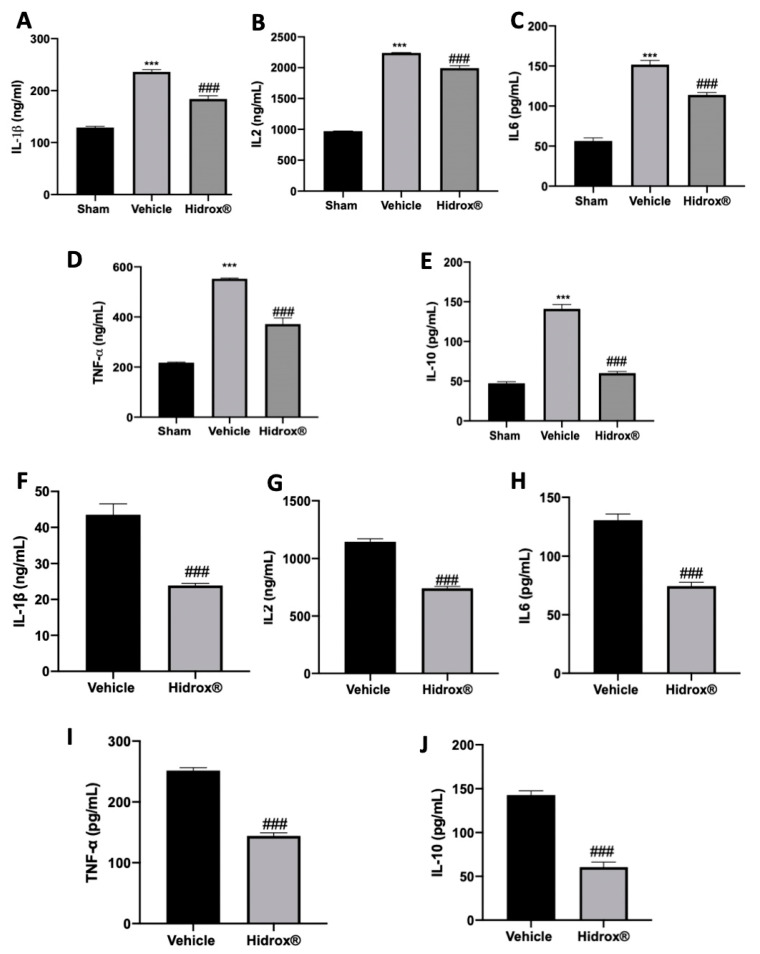
Hidrox^®^ administration reduced levels of cytokines: IL-1β (**A**), IL2 (**B**), IL6 (**C**), TNF-α (**D**), IL-10 (**E**) levels in peritoneal fluid. IL-1β (**F**), IL2 (**G**), IL6 (**H**), TNF-α (**I**) and IL-10 (**J**) levels in endometriotic lesions. For the analyses, *n* = 5 animals from each group were employed. A *p*-value of less than 0.05 was considered significant. *** *p* < 0.001 vs. sham, ### *p* < 0.001 vs. vehicle.

It has been described that in patients affected by endometriosis, peritoneal fluid directly reflects the changes of the local microenvironment. Thus, inflammatory cytokines in peritoneal fluid were focused. In peritoneal fluid of Hidrox^®^ treated rats, the levels of IL-1β (Figure 5A), IL2 (Figure 5B), IL6 (Figure 5C), TNF-α (Figure 5D) and IL-10 (Figure 5E) were increased as compared to the sham animals. Moreover, the analysis conducted on the lesions confirmed the same trend: elevated levels of IL-1β (Figure 5F), IL2 (Figure 5G), IL6 (Figure 5H), TNF-α (Figure 5I) and IL-10 (Figure 5J) were detected. Hidrox^®^ administration reduced in both peritoneal fluid and lesions reduced IL-1β (Figure 5A,E), IL2 (Figure 5B,F), IL6 (Figure 5C,G), TNF-α (Figure 5D,H) and IL-10 (Figure 5E,J) levels. 

### 3.6. Effect of Hidrox^®^ Treatment on Pain Sensitivity Threshold

**Figure 6:**
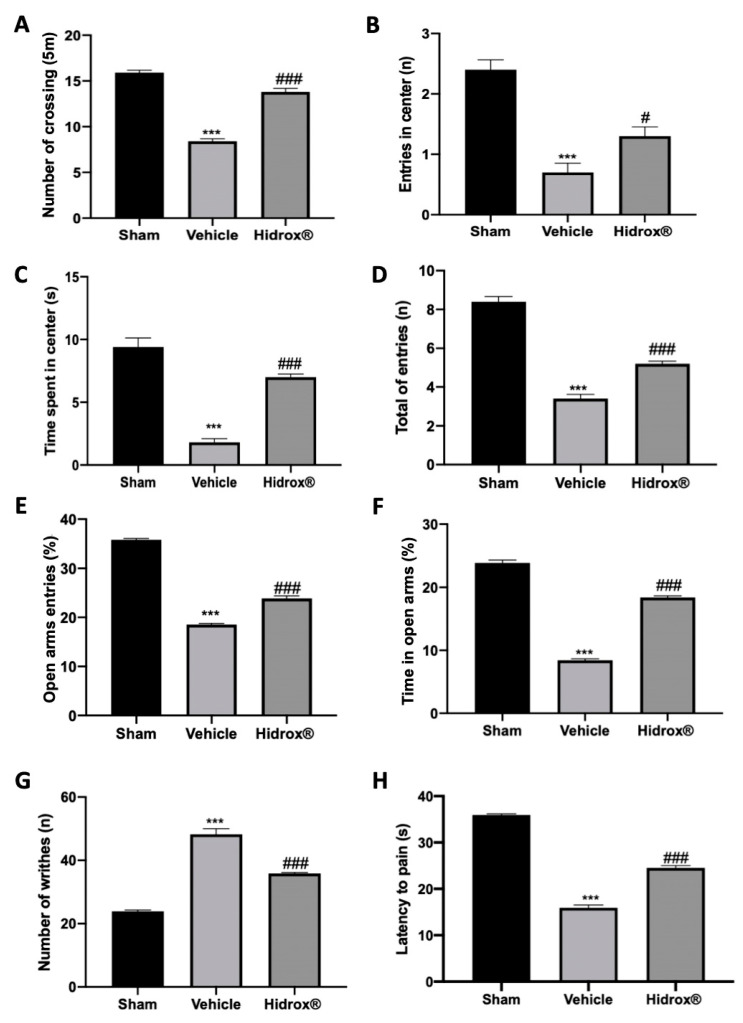
Hidrox^®^ administration reduced behavioral alterations endometriosis-induced: open field test: number of crossings (**A**), number of entries in central square (**B**), and time spent in central square (**C**); elevated plus maze test: number of entries in closed and open arms (**D**), % of open entries (**E**), % of time in open arms (**F**), acetic-acid-induced abdominal contractions (**G**), hot plate test (**H**). For the analyses, *n* = 5 animals from each group were employed. A *p*-value of less than 0.05 was considered significant. # *p* < 0.05 vs. vehicle, *** *p* < 0.001 vs. sham, ### *p* < 0.001 vs. vehicle.

Deep endometriosis induced behavioral alterations in vehicle-treated rats. In the open field test, vehicle-treated animals showed reduced spontaneous locomotion (Figure 6A), number of entries in the central square (Figure 6B) and time spent in it (Figure 6C). Hidrox^®^ treatment ameliorated locomotor activity and exploratory behavior (Figure 6A–C). In the elevated plus maze test, vehicle-treated rats showed reduced number of entries in closed and open arms (Figure 6D), % of open entries (Figure 6E) and the % of time in open arms (Figure 6F). Hidrox^®^ administration ameliorated all these parameters (Figure 6D–F). Vehicle-treated rats displayed a significant increase in the number of writhes, which were reduced by Hidrox^®^ treatment (Figure 6G). In the hot plate test, it was observed a significant reduction in the latency to pain reaction in the vehicle-treated, which was reduced by the Hidrox^®^ treatment (Figure 6H).

### 3.7. Effect of Hidrox^®^ Treatment on Tight Junctions Neuroinflammation

Tight junctions, mainly occludin and claudin-5, are important factors responsible for blood–brain barrier integrity. To further explore the impact on BBB integrity, we evaluated the level of occludin and claudin-5 by immunohistochemistry staining and Western blot.

**Figure 7:**
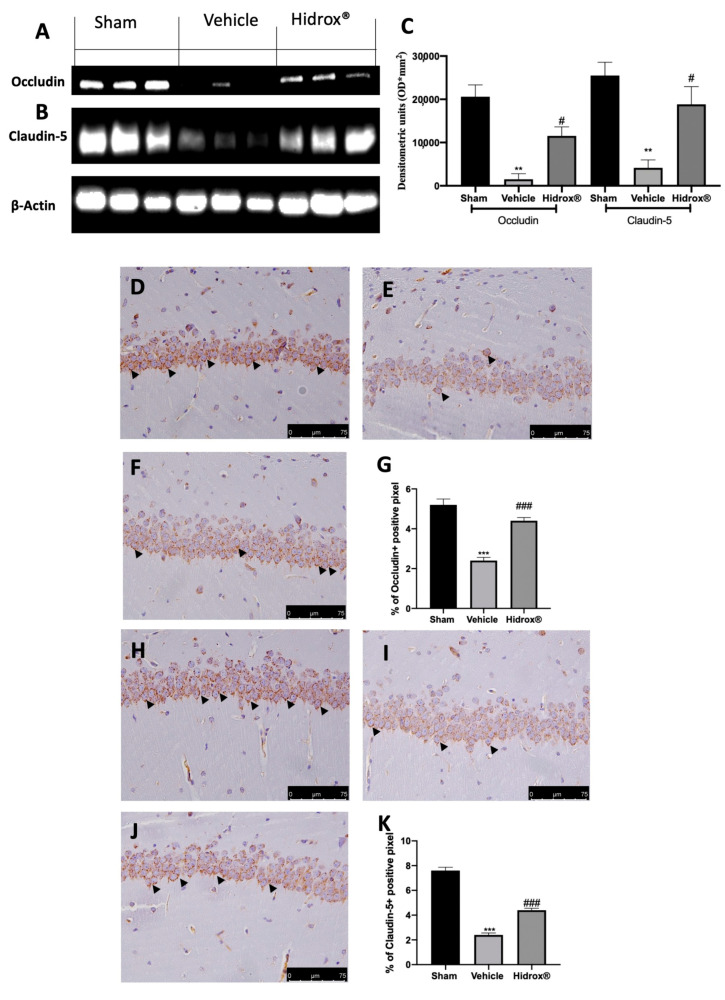
Hidrox^®^ administration partially restored tight junctions: Western blot analysis of occludin from hippocampal tissue (**A**), claudin-5 from hippocampal tissue (**B**), densitometric analysis (**C**), immunohistochemical analysis of occludin: sham (**D**), vehicle (**E**), Hidrox^®^ (**F**), graphical quantification of occludin expression (**G**); immunohistochemical analysis of claudin-5: sham (**H**), vehicle (**I**), Hidrox^®^ (**J**), graphical quantification of occludin expression (**K**). For the analyses, *n* = 5 animals from each group were employed. A *p*-value of less than 0.05 was considered significant. # *p* < 0.05 vs. vehicle, ** *p* < 0.01 vs. sham, *** *p* < 0.001 vs. sham, ### *p* < 0.001 vs. vehicle.

Tissues harvested from vehicle-treated rats showed increased blood–brain barrier permeability, as shown by the reduced expression of occludin (Figure 7A,C) and claudin-5 (Figure 7B,C). Hidrox^®^ administration partially restored both expression levels (Figure 7A–C). Immunohistochemical analysis revealed the same trend. Basal expression of occludin (Figure 7D,G) and claudin-5 (Figure 7H,K) was detected in sham animals, while vehicle-treated rats displayed reduced occludin (Figure 7E,G) and claudin-5 (Figure 7I,K) levels. Hidrox^®^ administration partially restored both occludin (Figure 7F,G) and claudin-5 (Figure 7J,K) levels. 

### 3.8. Effect of Hidrox^®^ Treatment on Neuroinflammation

**Figure 8:**
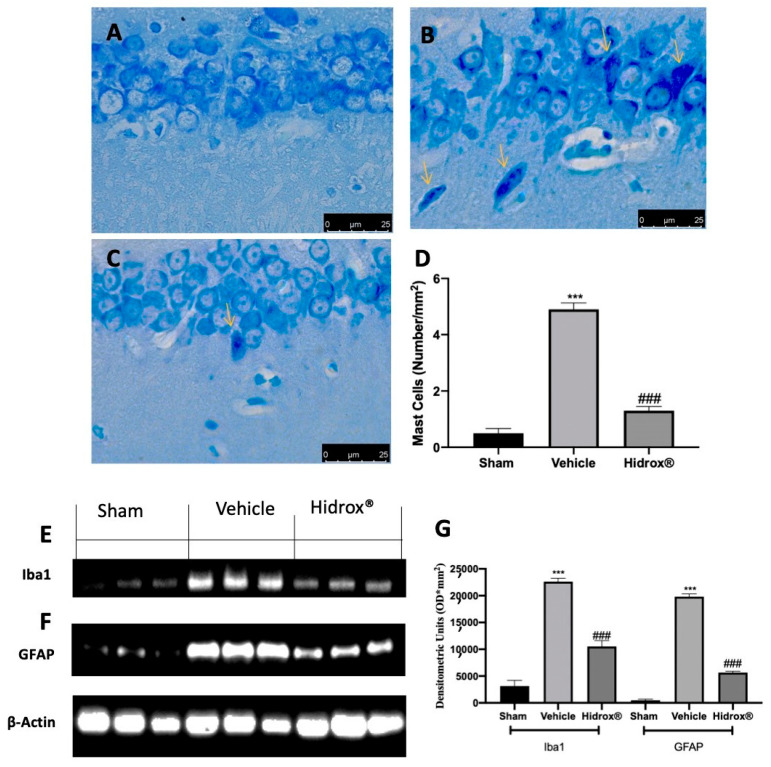
Hidrox^®^ administration reduced neuroinflammation endometriosis-induced: toluidine blue staining of hippocampal tissue: sham (**A**), vehicle (**B**), Hidrox^®^ (**C**), mast cell number (**D**), Western blot analysis of Iba from hippocampal tissue (**E**), GFAP from hippocampal tissue (**F**), densitometric analysis (**G**). For the analyses, *n* = 5 animals from each group were employed. A p-value of less than 0.05 was considered significant. *** *p* < 0.001 vs. sham, ### *p* < 0.001 vs. vehicle.

Further, it has been shown that a significant increase in mast cell number in the hippocampus is a sensor of brain injury and related to the stress-mediated neuroinflammation. Thus, we evaluated mast cell infiltration and Iba1 and GFAP expression. Toluidine blue staining showed increased mast cell infiltration in hippocampi from vehicle-treated rats (Figure 8B,D), as compared to the sham rats (Figure 8A,D). Animals treated with Hidrox^®^ showed reduced mast cell infiltration (Figure 8C,D). Western blot analysis showed a significant increase in Iba1 (Figure 8E,G) and GFAP (Figure 8F,G) expression in hippocampi tissues from vehicle-treated rats as compared to the sham tissues. Tissues harvested from Hidrox^®^-treated rats showed reduced expression of both neuroinflammatory markers (Figure 8E–G).

### 3.9. Effect of Hidrox^®^ Treatment on Oxidative Hippocampal Alterations

**Figure 9:**
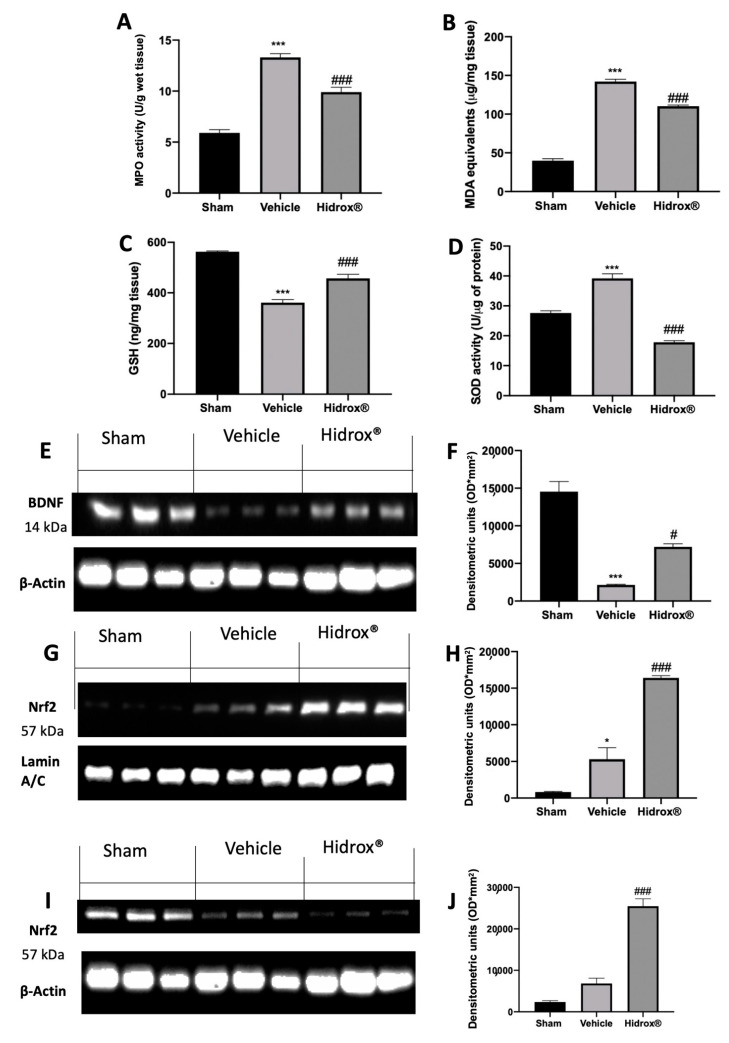
Hidrox^®^ administration reduced oxidative stress and improved BDNF and Nrf2 protein levels in hippocampus: MPO activity (**A**), MDA levels (**B**), GSH levels (**C**), SOD activity (**D**). Western blot analysis of BDNF from hippocampal tissue (**E**), densitometric analysis (**F**), Nrf2 nuclear expression from hippocampal tissue (**G**), densitometric analysis (**H**), Nrf2 cytosolic expression from hippocampal tissue (**I**), densitometric analysis (**J**). For the analyses, *n* = 5 animals from each group were employed. A *p*-value of less than 0.05 was considered significant. * *p* < 0.05 vs. sham, # *p* < 0.05 vs. vehicle, *** *p* < 0.001 vs. sham, ### *p* < 0.001 vs. vehicle.

Some evidence described the impaired brain oxidative status of rats subjected to endometriosis. Levels of GSH and MDA and MPO and SOD activities were determined in hippocampi. Western blot analyses were performed to evaluate BDNF and Nrf2 expressions. Vehicle-treated rats showed a significant increase in MPO activity and lipid peroxidation as compared to the sham animals. Treatment with Hidrox^®^ resulted in a significant reduction of MPO activity (Figure 9A) and MDA levels (Figure 9B). Moreover, Hidrox^®^ administration increased GSH levels (Figure 9C) and SOD activity (Figure 9D), as compared to the vehicle-treated rats. Western blot analysis showed a significant decrease in BDNF protein levels compared to the sham animals. Hidrox^®^ administration restored its expression (Figure 9E,F). Moreover, hippocampi from Hidrox^®^-treated animals showed increased Nrf2 nuclear translocation compared to the tissues harvested from vehicle-treated rats (Figure 9G,H). Differently, cytosolic expression of Nrf2 was decreased by Hidrox^®^ administration as compared with vehicle-treated rats (Figure 9I,J). 

## 4. Discussion

To our best knowledge, the induction and progression of endometriosis requires a proinflammatory environment, increased angiogenesis, changes in the epigenetic and structural elements and oxidative stress [60,61,62]. 

In particular, the oxidative damage, the elevation of inflammatory cytokines and the mast cell activation are considered a decisive step in the pathophysiology of endometriosis [63]. 

Our study demonstrated that Hidrox^®^ effectively decreased cyst diameter, area and volume and modified cyst morphology.

Advanced endometriotic lesions are characterized by widespread adhesions and fibrosis associated with pelvic morbidity, such as chronic pelvic pain and infertility [64]. Hidrox^®^ administration reduced collagen deposition and α-sma-positive myofibroblast in lesional stroma near the glandular epithelium showing reduced fibrosis. Angiogenesis is assumed to be a prerequisite for the formation and development of endometriosis [65,66]. Our results show that Hidrox^®^ treatment caused a reduction in CD34 and VEGF expression in the implants. Moreover, Hidrox^®^ administration was able to manage hyperproliferation and apoptosis during endometriosis. Many researchers found Ki67 and Bcl-2 overexpression and Bax downregulation in the gland and stroma of endometriotic loci [67]. Hidrox^®^ administration counteracted hyperproliferation and restored the apoptotic pathway in the lesions. From the histological point of view, mast cell involvement in endometriosis is well described [68,69]. The reduced number and degranulation of mast cells in endometriotic lesions from the Hidrox^®^-treated group relate to suppressed neuropathic pain and release of inflammatory mediators [70]. 

Several papers displayed a significant suppression of the production of the antioxidant defense, such as SOD activity, and increment of oxidized lipoproteins in the peritoneal microenvironment in women with endometriosis [71,72]. This rise in SOD activity occurred in response to the oxidative stress and to the high amount of ROS as an adaptive cell reaction accompanied by the decreased GSH and increased MDA levels. In this inflammatory condition, the innate immune system activates the phagocytic cells as shown by the increased MPO activity in the endometriosis rats [73]. MPO is a key enzyme of the innate immune system that produces oxidant radicals that can covalently alter proteins and lipids [73]. Indeed, Hidrox^®^ treatment by its antioxidant activity normalized the imbalance in oxidant–antioxidant activity in endometriotic rats as shown by the restored GSH levels, decreased SOD and MPO activity and lipid peroxidation. 

Studies conducted on patients with endometriosis showed increased levels of cytokines in both peritoneal fluid and ectopic lesions [74]. Anti-inflammatory cytokines are a class of immunoregulatory molecules that regulate the development of pro-inflammatory cytokines. Anti-inflammatory cytokines limit the potentially harmful effects of prolonged or excessive inflammatory responses under physiological conditions. Anti-inflammatory mediators in immune-mediated diseases may have inadequate control over pro-inflammatory behaviors under pathological conditions, or they may overcompensate and suppress the immune response, raising the risk of systemic infection. IL-6, IL-1β and TNF-α support adhesion of endometrial cells to the peritoneum, and TNF-α stimulates the proliferation of ectopic tissue. IL-4 and IL-10 family proteins are the main Th2 anti-inflammatory cytokines. Several lines of evidence indicate that the Th2 immune response is associated with endometriosis [75,76]. Some evidence demonstrated that anti-inflammatory cytokines, in particular IL-10 [77,78], are sharply increased in peritoneal fluid and the ectopic endometrium of women with endometriosis. Hidrox^®^ administration reduced levels of IL-2, IL-1β, TNF-α, IL-6 and IL-10 in the peritoneal fluid and endometriotic loci compared to the vehicle-treated group. Thus, Hidrox^®^ treatment significantly restored the pro-inflammatory microenvironment. 

Our experimental conditions showed an interesting relationship between the growth of the implants, the inflammatory microenvironment and the development of pain-like symptoms. As stated previously, a severe local inflammatory and hemorrhagic response occurs at the beginning of the endometriosis establishment. In particular, inflammation grows exponentially with the size of the cyst and invasion of peritoneal organs [79].

It was recently shown that endometriosis exacerbated inflammatory manifestations and altered pain threshold [80]. In the present paper, we investigated rat pain perception based on different tests for peripheral and visceral sensibility, respectively, at the development of the endometriosis model. In particular, in accordance with literature showing decreased mechanical and thermal pain threshold in rats and mice in a similar model of endometriosis [81,82], our results show that endometriosis rats presented increased visceral sensitivity. Animals subjected to endometriosis and treated with Hidrox^®^ displayed reduced thermal and mechanical hyperalgesia and pain sensitivity. 

Regarding vulnerability to pain, endometriosis is related to central and peripheral pain sensitization [83]. Initially, tissue injury and inflammation sensitized in the peripheral nociceptive system, producing a decreased pain threshold and an increased sensory input to the central nervous system. With these continuous stimuli, because of long-term central adaptations in the process called central sensitization, central behavior may become independent of any peripheral inputs [84]. One of the crucial brain regions involved in the affective and cognitive consequence of neuropathic pain is the hippocampus [85]. Patients with endometriosis displayed abnormal connectivity in the hippocampus and in their afferences to the frontoinsular and somatosensory cortex [86]. This brain area, in fact, is involved in the transition from acute to chronic pain [87]. Further, it has been reported that a significant increase in mast cell number in the hippocampus is a sensor of brain injury and related to the stress-mediated neuroinflammation [88,89]. Mast cells act by amplifying neuroinflammation through microglial and astrocytes activation [90]. They are the resident immune cells in the brain and play a pivotal role in immune surveillance of the central nervous system (CNS).

Whereby the mechanism employed by mast cells to transit the brain capillary endothelium remains to be fully characterized, several investigations support the hypothesis of the disruption of the blood–brain barrier [91]. Well in line with the literature, we observed occludin and claudin-5 protein changes in the brain harvested from the endometriosis rats. Additionally, our findings show increased mast cell degranulation and Iba1 and GFAP expression in endometriosis rat hippocampus. Hidrox^®^ treatment was able to reduce degradation of tight junction proteins and mast cell infiltration in the hippocampus. Therefore, Hidrox^®^ reduced microglial and astrocytes activation.

Additionally, ROS overproduction by microglia is suggested to be a main cause of neuronal damage and dysfunction [92,93,94] inducing derangement of neuronal redox signaling circuits or direct oxidative damage [95,96]. Multiple evidence supports the role of oxidative stress in the progress of endometriosis [13,14,97]. A recent study evaluated the brain oxidative status of rats subjected to endometriosis [98]. In particular, oxidative alterations in the hippocampus of endometriosis rats have been described. Here, we displayed that endometriosis induced a pronounced oxidative imbalance in rat hippocampus, as evidenced by the reduced levels of the endogenous antioxidant GSH, the increased SOD activity and the augmented lipid peroxidation. 

Additionally, the induction of an immune oxidative environment in the endometriosis rat hippocampus is one of the underlying mechanisms of the pain sensitization observed. Hidrox^®^ administration was able to restore the oxidative balance in rat hippocampus subjected to endometriosis by managing GSH levels, SOD and MPO activity and lipid peroxidation. We propose that Hidrox^®^ antioxidant and anti-inflammatory activity would contribute to the reduced pain-like symptoms.

In accordance with these immuno-oxidative findings, it has been demonstrated that an increased oxidative status in the hippocampus is related to reduced BDNF levels [99]. It is a neurotrophic factor that regulates synaptic plasticity and brain neurogenesis. Moreover, BDNF controls the Nrf2 translocation into the nucleus, which is a transcription factor responsible for the activation of several antioxidant defenses [100]. Hidrox^®^ treatment by reducing the persistent state of oxidative stress in the hippocampus of endometriosis animals was able to restore BDNF levels which would be a central mechanism underlying the pro-oxidative and behavioral changes in endometriosis. As already described [25,101], Hidrox^®^ increased Nfr2 nuclear translocation which in turn activates several genes with cytoprotective function restoring the redox homeostasis.

## 5. Conclusions

In conclusion, our results show that Hidrox^®^ is a very effective antioxidant and a powerful anti-inflammatory agent. Therefore, our hypothesis is that Hidrox^®^ carries out its action through the modulation of the oxidant/anti-oxidant balance, the reduction of the hyperproliferation and angiogenesis leading to smaller lesion sizes. Hidrox^®^ administration by restoring oxidative balance in the hippocampus, a crucial mood-regulating region of the brain, also involved in the processing of nociception, relieves endometriosis-associated pain.

## Data Availability

The data presented in this study are available on request from the corresponding author.
