# Correction: Cordaro et al. Hidrox^®^ and Endometriosis: Biochemical Evaluation of Oxidative Stress and Pain. *Antioxidants* 2021, *10*, 720

**DOI:** 10.3390/antiox14091030

**Published:** 2025-08-22

**Authors:** Marika Cordaro, Angela Trovato Salinaro, Rosalba Siracusa, Ramona D’Amico, Daniela Impellizzeri, Maria Scuto, Maria Laura Ontario, Livia Interdonato, Roberto Crea, Roberta Fusco, Salvatore Cuzzocrea, Rosanna Di Paola, Vittorio Calabrese

**Affiliations:** 1 Department of Biomedical, Dental and Morphological and Functional Imaging University of Messina, Via Consolare Valeria, 98125 Messina, Italy; cordarom@unime.it (M.C.); dipaolar@unime.it (R.D.P.); calabres@unict.it (V.C.); 2 Department of Biomedical and Biotechnological Sciences, University of Catania, 95124 Catania, Italy; trovato@unict.it (A.T.S.); mary-amir@hotmail.it (M.S.); marialaura.ontario@ontariosrl.it (M.L.O.); 3 Department of Chemical, Biological, Pharmaceutical and Environmental Sciences, University of Messina, 98166 Messina, Italy; rsiracusa@unime.it (R.S.); rdamico@unime.it (R.D.); dimpellizzeri@unime.it (D.I.); livia.interdonato@yahoo.it (L.I.); 4 Oliphenol LLC., 26225 Eden Landing Road, Unit C, Hayward, CA 94545, USA; robertocrea48@gmail.com

In the original publication [1], there was an error in Figure 4B. The authors unintentionally included some incorrect figures. In detail, in Figure 4, in the group Hidrox, they inadvertently attached the wrong picture. 

The authors checked all the data in their laboratory, found the original photos, and prepared a revised figure using an appropriate representative image from their database, belonging to the experimental groups in question.

The authors apologize for any inconvenience caused by this oversight. The new Figure 4 appears below. The authors state that the scientific conclusions are unaffected. This correction was approved by the Academic Editor. The original publication has also been updated.

## Figures and Tables

**Figure 4:**
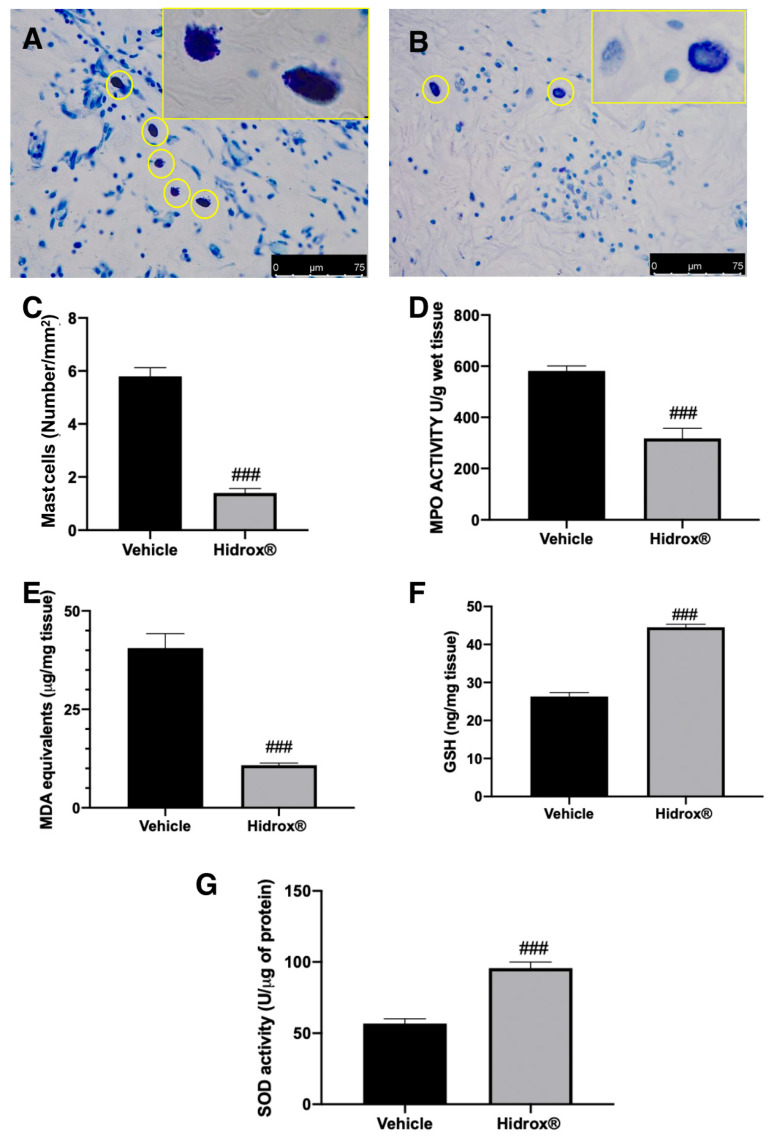
Hidrox^®^ administration reduced mast cell number and pro-oxidative alterations in endometrial explants: Toluidine blue staining of explanted lesions: vehicle (**A**), Hidrox^®^ (**B**), mast cell number (**C**), MPO activity (**D**), MDA levels (**E**), GSH levels (**F**), SOD activity (**G**). For the analyses, *n* = 5 animals from each group were employed. A *p*-value of less than 0.05 was considered significant. ### *p* < 0.001 vs. vehicle.
